# Hierarchical Composite Meshes of Electrospun PS Microfibers with PA6 Nanofibers for Regenerative Medicine

**DOI:** 10.3390/ma13081974

**Published:** 2020-04-23

**Authors:** Zuzanna J. Krysiak, Małgorzata Z. Gawlik, Joanna Knapczyk-Korczak, Łukasz Kaniuk, Urszula Stachewicz

**Affiliations:** International Center of Electron Microscopy for Material Science, Faculty of Metals Engineering and Industrial Computer Science, AGH University of Science and Technology, 30-059 Cracow, Poland; krysiak@agh.edu.pl (Z.J.K.); mg.gawlik@gmail.com (M.Z.G.); jknapczyk@agh.edu.pl (J.K.-K.); kaniuk@agh.edu.pl (Ł.K.)

**Keywords:** polystyrene, nylon 6, electrospun fibers, composite mesh, proliferation, roughness

## Abstract

One of the most frequently applied polymers in regenerative medicine is polystyrene (PS), which is commonly used as a flat surface and requires surface modifications for cell culture study. Here, hierarchical composite meshes were fabricated via electrospinning PS with nylon 6 (PA6) to obtain enhanced cell proliferation, development, and integration with nondegradable polymer fibers. The biomimetic approach of designed meshes was verified with a scanning electron microscope (SEM) and MTS assay up to 7 days of cell culture. In particular, adding PA6 nanofibers changes the fibroblast attachment to meshes and their development, which can be observed by cell flattening, filopodia formation, and spreading. The proposed single-step manufacturing of meshes controlled the surface properties and roughness of produced composites, allowing governing cell behavior. Within this study, we show the alternative engineering of nondegradable meshes without post-treatment steps, which can be used in various applications in regenerative medicine.

## 1. Introduction

The vastly growing field of regenerative medicine is continuously looking for new materials and novel ways to improve currently used materials for cell culture studies. Many studies showed the importance of cells–materials interactions and how to modify the surface to enhance cell adhesion and proliferation, which are responsible for tissue growth [1,2,3]. In regenerative medicine, one of the most frequently used polymers is polystyrene (PS), which is hydrophobic and thus, often, its surface needs modification by entering hydroxyl groups to achieve hydrophilic behavior [4]. This modified PS is a so-called tissue culture polystyrene (TCPS) that enables easy cell attachment and proliferation. Therefore, it is widely used in cell culture experiments in the form of a flat surface [5,6]. Films, which are 2D structures, are mostly applied for in vitro tests, whereas for tissue engineering, 3D porous constructs are more preferable for cell development. A variety of methods can be applied for 3D meshes manufacturing [7]. Those produced via electrospinning have a structure with high porosity and a high surface area to volume ratio [8,9]. The porosity can be controlled via fiber size governed by electrospinning parameters, as well as the application of various collectors [10]. This technique allows the fabrication of both random and aligned fibers for different applications with a wide range of sizes from nano to micrometers, which influence different cell behavior on manufactured material [7,11,12,13]. Aligned fibers fabricated via electrospinning were applied to build the ligament tissue based on the hierarchical structure [14]. A combination of nano with micro electrospun fibers in the scaffold was found to be a promising material for bone tissue regeneration [15]. A two-nozzle electrospinning set-up [16] can be used to obtain composite structures [17] made of nano and microfibers [18].

Polyamides are commercially used as surgical struts [19], in many cardiovascular applications [20], and also for the production of artificial tendons, ligaments, joints [21] and inguinal meshes [22]. PS with nylon 6 (PA6) is known for its high mechanical strength, biocompatibility, flexibility, and similarity to the peptides concerning amide bonds. Electrospun PA6 fibers were blended with other polymer fibers [23,24], which resulted in increased cell proliferation [25,26] applied in the wound and burn treatment [27]. Additionally, the wetting behavior of PA6 fibers can be controlled via electrospinning itself [28,29], and the wetting properties of a material are crucial factors in biomedical applications. Both the chemistry in the meaning of hydrophobicity or hydrophilicity and roughness influence surface wettability [30,31].

PS is mostly used for in vitro studies [4,5,32], but without any surface modification, for example, sliver negative ion implementation [33], protein absorption [34], or plasma treatment [35,36], does not enhance cell development. PS fibers have been already combined with PA6 fibers [37] in the fog collector’s meshes, comparing fiber diameter, roughness, contact angle, and the showing mechanical properties of PS, PA6, and PS-PA6 mats of maximum stress 0.03, 1.24 and 0.07 MPa, respectively [38]. Similar designs of nondegradable polymers have the potential to be used in vascular tissue engineering [39] or hernia meshes [40,41]. Moreover, electrospinning was often used to produce highly porous materials with controlled morphology and mechanical properties for vascular grafts [42].

Therefore, the goal of this study was to electrospin hierarchical constructs containing PS in the form of microfibers with the addition of hydrophilic PA6 nanofibers to produce 3D structures. To fabricate our meshes, we applied a two-nozzle system to electrospun both polymers at the same time, aiming towards the biomimetic extracellular matrix (ECM) in terms of wetting and roughness [43]. Importantly, we showed that the chemical or oxidation modifications of hydrophobic PS modification can be replaced by adding hydrophilic PA6 nanofibers into meshes. The engineered hierarchical and fibers-based composite meshes can be applied in regenerative medicine to control cell behavior and to firmly integrate with living tissue.

## 2. Materials and Methods

### 2.1. Solutions Preparation

Prior to the solution’s preparation, polymers were dried in an oven (SLN32STD, POL-EKO-APARATURA sp.j., Wodzisław Śląski, Poland) for 3 h at T = 30 °C. PS (Sigma Aldrich, Gillingham, UK, Mw = 350,000 g∙mol^−1^) was dissolved in dimethylformamide (DMF, 99.8%, POCH, Gliwice, Poland) at a concentration of 25 wt%, PA6 (BASF, Ludwigshafen, Germany, Mw = 24,000 g·mol^−1^) was dissolved in formic acid (85%, POCH, Gliwice, Poland), and acetic acids (99.5%, POCH, Gliwice, Poland) mixed in a volume ratio of 1:1 at 12 wt%. Both solutions were stirred at 500 rpm for 4 h at 20 °C (IKA RCT basic, Staufen, Germany). The concentration of the polymer solution was adjusted according to the molecular weight of the polymer to obtain suitable viscosity, allowing it to produce beadles’ fibers.

### 2.2. Electrospinning and Meshes Characterization

**Figure 1:**
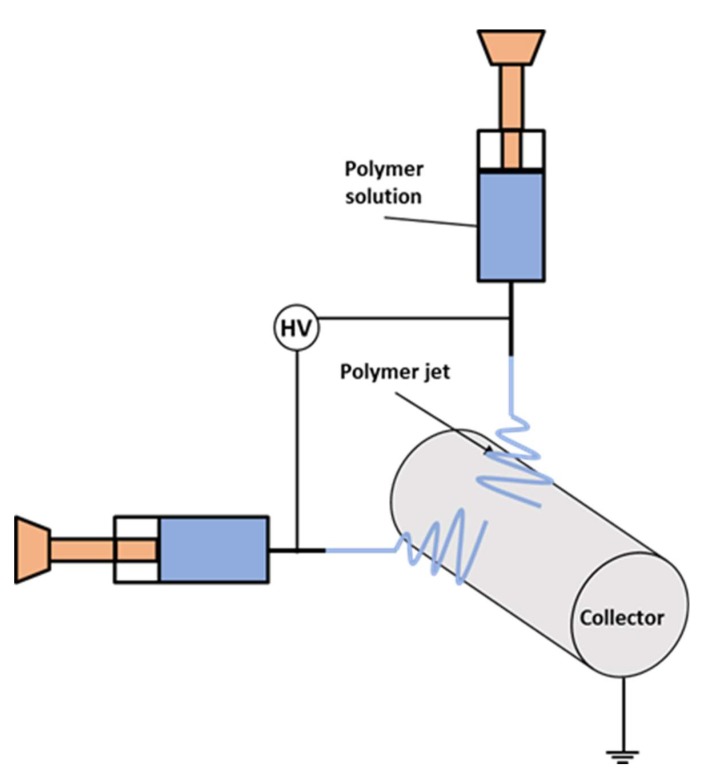
The electrospinning set-up consisting of two nozzles to produce polystyrene (PS) and PS with nylon 6 (PA6) fibers at the same time to obtain the composite meshes.

Fibers were electrospun using a set-up with the climate-controlled chamber (IME Technologies, Waalre, The Netherlands) at T = 25 °C and H = 40%, then deposited on the slowly rotating (10 rpm) collector covered with an Al foil; see the schematic in Figure 1. Three different fiber mats were produced: PS, PA6, and PS-PA6. For the first two mats, only one nozzle was used. High voltage with positive voltage polarity in the range of 13–20 kV was applied to the needle kept at a distance of 15–22 cm from the collector, with all the other parameters listed in Table 1. The quality of the electrospun fibers was analyzed with the scanning electron microscope (SEM, Merlin Gemini II, Zeiss, Oberkochen, Germany) after the samples were sputtered with gold (Q150RS, Quorum Technologies, Laughton, UK). The diameters of the fibers were measured using ImageJ (v1.51s, USA), and the average fiber diameter was calculated from 100 measurements in the SEM images. Additionally, the contact angles were measured on the electrospun mats using deionized water. Pictures of water droplets were taken with a Canon EOS 700D camera (Tokyo, Japan) with an EF-S 60 mm f/2.8 Macro USM zoom lens 3 s after placing the 3-µL droplets on the mats. The contact angles were measured using MB-Ruler (version 5.3, Germany) based on the sessile drop method and the mean value was calculated as the average of 10 droplets. The roughness of the electrospun samples was analyzed in our previous reports [38], where a laser microscope (Olympus OLS4000, Tokyo, Japan) was used. Prior to the roughness analysis, the electrospun samples were deposited on glass slides and covered with the 5-nm gold layer. Ten measurements per sample type were performed, with the investigated area of 646 × 646 μm^2^ for PS and PS-PA6 composites, and 130 × 130 μm^2^ for PA6.

### 2.3. Cell Culture Studies

NIH 3T3 murine fibroblast cells (Sigma Aldrich, Gillingham, UK) were used for proliferation and adhesion assay on the meshes sterilized with UV light. Cells were seeded on PS, PA6, and PS-PA6 composite fibers, and on the bottom of a 24-well plate (TCPS) as a reference, with a concentration of 2 × 10^4^ cells per sample. Samples with cells were incubated at H = 90%, T = 37 °C, and CO_2_ set to 5% up to 7 days. Cell proliferation and adhesion assays were performed using MTS (CellTiter 96^®®^ AQueous One Solution Cell Proliferation Assay, MTS, Promega, Madison, WI, USA). The cell morphology was examined with SEM. NIH 3T3 cells were cultured in Dulbecco’s Modified Eagle Medium (DMEM with 4.5 g/L D-Glucose, Gibco, Paisley, UK), supplemented with 10% of Fetal Bovine Serum (FBS, Sigma Aldrich, St. Louis, MO, USA), 1% of L-Glutamine solution (Sigma Aldrich, St. Louis, MO, USA), 2% of antibiotics (Penicillin-Streptomycin, Sigma Aldrich, St. Louis, MO, USA), and 1% of amino acids (Mem non-essential Amino Acid solution 100x, Sigma Aldrich, St. Louis, MO, USA). The medium was changed three times a week.

#### 2.3.1. Adhesion Test

Adhesion assay was performed 1.5, 2, and 4 h after cell seeding. Samples were washed with PBS prior to MTS reagent, added to rinse unattached cells. Next, 80 µL MTS reagent and 400 µL of fresh cell culture medium were added to each sample and reference. It was incubated for 4 h at 37 °C, H = 90% and a CO_2_ concentration of 5%. After that time, 100 µL of the reaction solution from every sample was transferred to a 96-well plate in triplicates. The absorbance at a wavelength of 490 nm was measured using a Microplate Reader (LT-4000, Labtech, Aylesbury, UK).

#### 2.3.2. MTS Proliferation Assay

Proliferation was assessed after 1, 3, and 7 days of fibroblast culture. After each time point, the culture medium was removed, and meshes were transferred to the new 24-well plate. Then, 80 µL of MTS reagent and 400 µL of cell culture medium were added, and assay was proceeded as described above. For proliferation assessment with SEM, after the defined time of 1, 3, and 7 days, cell culture samples were transferred to the new 24-well plate and rinsed 3 times with PBS. Next, they were fixed with a 2.5% formaldehyde solution for 2 h at 4 °C. The solution was removed and the samples were again rinsed with PBS solution, then dehydrated using a series of ethanol solutions with the concentrations: 50%, 70%, 96%, and 99.9%. The meshes were left to dry under the hood. Before the SEM observation, the samples were coated with a 5 nm layer of Au.

#### 2.3.3. Statistical Analyses

The statistical analyses were performed using OriginPro (v2019 SR2, OriginLab, USA). Analysis of variance (ANOVA) with a Tukey test was performed with significance at p < 0.02. For fiber diameter and contact angle measurement, errors are based on standard deviation calculation.

## 3. Results and Discussion

### 3.1. Fibers Characterization

**Figure 2:**
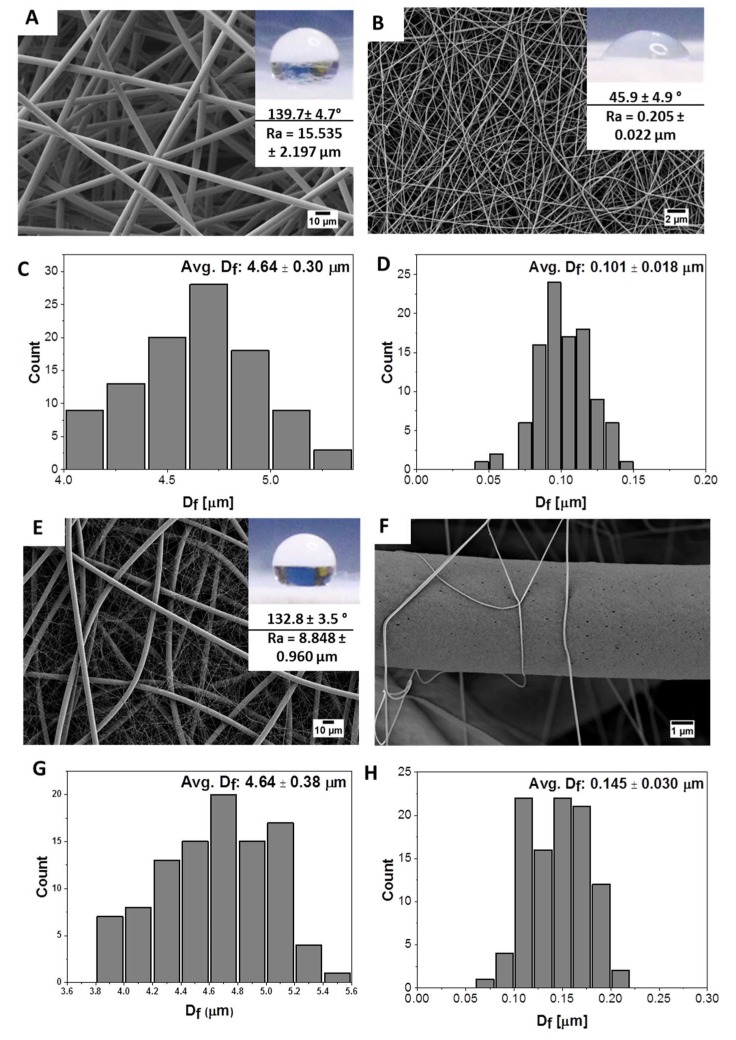
SEM micrographs of electrospun fibers of (**A**) PS, (**B**) PA6, (**E**) PS-PA6 composites, and (**F**) PS-PA6 composite with the higher magnification showing a few PA6 nanofibers on the individual PS microfiber. The fiber diameter distribution showed in histograms for (**C**) -PS; (**D**) -PA6, (**G**) -PS in the composite, and (**H**) -PA6 in the composite meshes.

Prior to the cell culture study, the quality and morphology of the produced electrospun fibers were verified with SEM; see Figure 2. The average fiber diameter for PS was 4.62 ± 0.3 µm (Figure 2C) and 0.101 ± 0.018 µm for PA6 (Figure 2D). The size of PS fibers was similar in the fibrous composite. However, the average fiber diameter of PA6 fibers was increased to 0.145 ± 0.030 µm, due to the slight adjustment of the electrospinning parameters; see Table 1 and Figure 2G,H, as previously described [44]. The water contact angle measurement confirmed PA6 hydrophilicity (45.9 ± 4.9°) and PS (139.7 ± 4.7°) hydrophobicity and also showed the hydrophobic character of produced composite (132.8 ± 3.5°); see Figure 2. According to the previous study, the larger the average fiber diameter, the higher the surface roughness (R_a_), which reached 15.535 ± 2.197 µm for PS, 0.205 ± 0.222 µm for PA6, and 8.848 ± 0.960 µm for PS-PA6 [38]. Additionally, R_a_ strongly influences the wetting behavior of electrospun membranes [31]. Here, the hydrophobic character was obtained for PS-PA6 composite mesh mainly due to the roughness effect [44], and the water droplets still kept the contact points with PS fibers. The presence of PS and PA6 fibers was already confirmed by the X-ray photoelectron microscopy analysis (XPS) reported in our studies previously [38], where also the roughness and water contact angle were investigated according to PA6 content. Based on the reported data in [38], the increased PA6 fraction of nanofibers that usually forms a compact layer of membrane lowers the surface roughness once combined with PS microfibers. The increase in PA6 nanofibers fraction, controlled with a longer electrospinning time, decreased the roughness and water contact angle only slightly. Importantly, the PA6 meshes are characterized by relatively small pore sizes of 1.7 μm, and a very high porosity of 96% in meshes [45]. In addition to the morphology, the mechanical properties of manufactured PS-PA6 hierarchical composite meshes were also investigated in various configurations, showing higher tensile stress for PA6 (1.24 MPa) than for PS (0.3 MPa) fibers. The incorporation of PA6 fibers into PS meshes significantly improved the mechanical properties of composite meshes reaching 0.6 MPa [38].

### 3.2. Cell Culture Study

**Figure 3:**
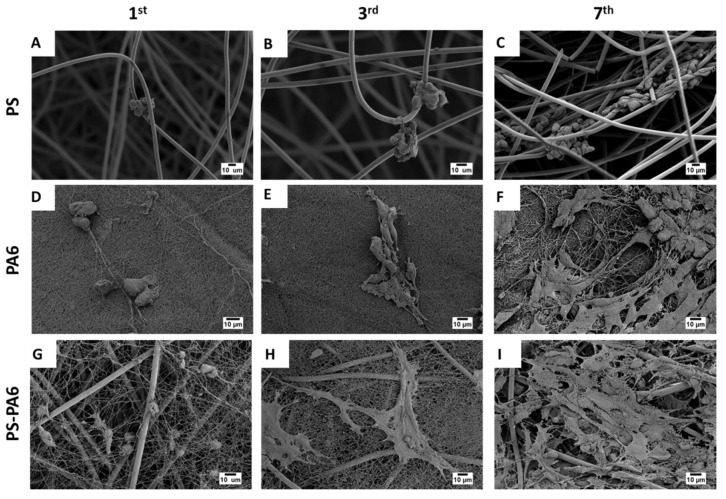
SEM micrographs showing fibroblasts growth on electrospun fiber after the 1st, 3rd, and 7th day in cell culture on (**A**–**C**) PS meshes, (**D**–**F**) PA6 meshes and (**G**–**I**) PS-PA6 composite meshes, respectively.

The SEM micrographs of fibroblast on electrospun fibers and composites shown in Figure 3 indicate a clear difference in cell behavior on the three types of tested meshes. After one day of incubation, cells started to attach to the fibers, but still kept spherical shapes (Figure 3A,D,G). The cells on the PS mats did not flatten even after 7 days of culturing (Figure 3C), in contrast to PA6 nanofibers and PS-PA6 composite meshes, where cells clearly started already spreading on the fibers after the third day. The cells’ attachment and spreading prove their integration with the mesh [15,46]. Importantly, by incorporating PA6 nanofibers to PS fibers, we decreased the surface roughness of meshes significantly, as the PA6 fibers were 100 nm in diameter [44]. Fibroblasts prefer a lower surface roughness [43,47] for spreading and migration [48]. Additionally, the hydrophilic character of PA6 fibers leads to evident cell spreading and attachment, causing further enhanced cell development [30,31]. The SEM observations, shown in Figure 3 prove that PS-PA6 composite meshes were enhancing cell flattening and proliferation.

**Figure 4:**
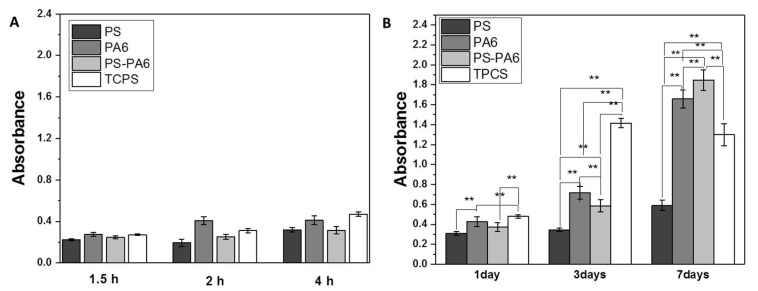
Cell culture study on electrospun PS, PA6 and PS-PA6 composite meshes showing (**A**) the adhesion test: 1.5 h, 2 h, and 4 h and (**B**) proliferation assay after 1, 3 and 7 days after cell seeding. ** statistical significance calculated with ANOVA, followed by Tukey’s post-hoc test, p < 0.02, error bars are based on standard deviation.

**Figure 5:**
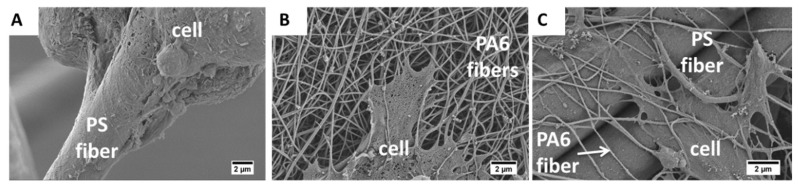
SEM micrographs focused on cell–fiber attachment after the 3rd day of cell culture on (**A**) PS microfibers; (**B**) PA6 nanofibers and (**C**) hierarchical PS-PA6 composite meshes.

The surface properties of meshes are essential for cell attachment. In the first few hours, the process of cell anchoring already begins, leading to further cell development and proliferation [49,50,51]. The adhesion test performed during the first 4 h of cell culture indicates no significant difference in cell attachment between all fibrous meshes and TCPS, see Figure 4A. Interestingly, cell geometry and attachment to PS, PA6, and PS-PA6 composite varied. In Figure 5, we show the SEM images focused on cell filopodia anchoring to fibers. Additionally, the shape of the created filopodia and the cell flattening were different between the three types of samples. The cells kept the round shape on PS fibers (Figure 5A) and PA6 nanofibers, but PS-PA6 composites were flattened (Figure 5B,C). Moreover, the changes in filopodia’s morphology defined totally different cells spreading, which is crucial for tissue regeneration and biomaterials integration with the living system. The further proliferation assay in Figure 4B shows no differences between materials after 1 day of cell culture. However, after the third day, the absorbance values started to increase for PA6, especially for the PS-PA6 composite, which continued up to the seventh day. The MTS test for PS meshes was close to constant over one-week of cell culture, indicating that the cell proliferation was only kept at a minimal level. Importantly, after 7 days, the number of cells on PS-PA6 composite meshes was greater than on any other tested samples and TCPS (Figure 4B). Even though the statistical analysis showed differences between materials after the same period, SEM images of cells also have to be taken into consideration to conclude which material has the most suitable properties for cell proliferation. It was previously shown that decreasing the surface roughness and hydrophobicity enhances cell proliferation [52]. In our study, the incorporation of PA6 nano-sized fibers into PS fibers decreased the roughness by half R_a,_ and only slightly decreased the water contact angle values; see Figure 2. However, cell proliferation was visibly higher, as presented in Figure 4B. Indeed, the decreased R_a_ and hydrophilic character of PA6 fibers enhanced cell proliferation. As Ansleme et al. described, the short-term adhesion and proliferation were more influenced by surface chemistry, while surface roughness affects long-term behavior [31]. We noticed a better adhesion for more hydrophilic materials, such as PA6 meshes or TCPS. The PS-PA6 composite meshes were characterized by a water contact angle above 130°, thus showing that hydrophobic behavior does not have a straightening effect. Noticeably, by adding PA6 to meshes, we also changed the surface chemistry by including the oxygen groups that were eventually detected by XPS [38]. This type of surface with increased oxygen content is preferable for cell adhesion [31]. Indeed, after one week of cell culture, the R_a_ decreased by half for PS-PA6 composite meshes in comparison to PS showed the highest value of absorbance for cell proliferation. Interestingly, the size of PS fibers in order of magnitude was higher than for PA6, with increased cell penetration into meshes and growth inside them, whereas in just PA6 meshes, the small distances between nanofibers were limiting cells to the top surface of the samples. The increase of PA6 fraction decrees the spacing between fibers as the smaller fiber diameter in electrospun random meshes cause the smaller distance between fibers [53]. The fiber diameters also control the roughness of meshes [44]. Therefore, the content of PA6 nanofibers was selected not to limit the cell’s integration with electrospun meshes. We showed previously on PMMA nanofibers, microfibers, ribbons, and films how the cell morphology is changing according to the surface topography. A diameter of fiber exceeding 3.5 µm is required to provide enough spacing for cell migration into the 3D meshes and enhance the filopodia attachments to fibers underneath [53]. The increased fraction of nanofibers facilitates more cell spreading on the top of the surface in comparison to the microfibers; therefore, in our PS-PA6 hierarchical scaffolds, the layering electrospinning providing a higher number of PA6 fibers were not investigated in vitro in this study.

## 4. Conclusions

Within this study, we were able to fabricate a hierarchically structured composite consisting of PS microfibers and PA6 nanofibers using a two-nozzle electrospinning set-up in the single-step manufacturing method. We showed that controlling the surface morphology, chemistry, and roughness of composite meshes guided fibroblasts behavior and development. The filopodia formation and their further proliferation were affected by the size of fibers. The micronized PS fibers allowed deeper penetration of cells, allowing enhanced material integration with the living systems. Moreover, nanosized PA6 fibers with hydrophilic wetting behavior by itself promoted cell development and spreading, despite the fact that the obtained composites were, in general, hydrophobic. Manipulating the rate of PA6 nanofibers in the PS network of fibers allows us to tailor mechanical properties [37] and roughness of meshes [38], thus designing the environment for desired cell types [30,39]. The proposed combination of polymers and its structure is leading to multiple application strategies of nondegradable meshes supporting tissue in regenerative medicine.

In summary, we introduced a novel way to produce PS-PA6 composite meshes in a single-step manufacturing method to enhance cell proliferation and development. These nondegradable polymer composite meshes are able to create a favorable environment for cells reminding ECM with a perspective use in regenerative medicine and for in vitro studies of disease models as 3D constructs.

## Figures and Tables

**Table 1:** Electrospinning parameters of PS, PA6, and with two nozzles spinning at the same time to produce PS-PA6 composite meshes.

Polymer	Voltage Applied to the Needle (kV)	Voltage Applied to the Collector (kV)	Distance Between Needle and Collector (cm)	Flow Rate (mL·h^−1^)
PS	13	0	15	1.5
PA6	18	−2	15	0.2
PS-PA6	20	0	22/17	1.8/0.1
